# Variations in abundance, diversity and community composition of airborne fungi in swine houses across seasons

**DOI:** 10.1038/srep37929

**Published:** 2016-11-28

**Authors:** Priyanka Kumari, Cheolwoon Woo, Naomichi Yamamoto, Hong-Lim Choi

**Affiliations:** 1 Department of Agricultural Biotechnology, Research Institute of Agriculture and Life Sciences, College of Agriculture and Life Sciences, Seoul National University, Seoul 151-921, South Korea; 2 Department of Environmental Health, Graduate School of Public Health, Seoul National University, Seoul 151-742, South Korea

## Abstract

We examined the abundance, diversity and community composition of airborne fungi in swine houses during winter and summer seasons by using quantitative PCR and Illumina HiSeq sequencing of ITS1 region. The abundance of airborne fungi varied significantly only between seasons, while fungal diversity varied significantly both within and between seasons, with both abundance and diversity peaked in winter. The fungal OTU composition was largely structured by the swine house unit and season as well as by their interactions. Of the measured microclimate variables, relative humidity, particulate matters (PMs), ammonia, and stocking density were significantly correlated with fungal OTU composition. The variation in beta diversity was higher within swine houses during summer, which indicates that the airborne fungal community composition was more heterogeneous in summer compared to winter. We also identified several potential allergen/pathogen related fungal genera in swine houses. The total relative abundance of potential allergen/pathogen related fungal genera varied between swine houses in both seasons, and showed positive correlation with PM2.5. Overall, our findings show that the abundance, diversity and composition of airborne fungi are highly variable in swine houses and to a large extent structured by indoor microclimate variables of swine houses.

The use of confinement buildings with high animal density is very common in modern animal husbandry. The concentrations of volatile organic compounds, ammonia (NH_3_), sulfide and particulate matters (PMs) are elevated in the indoor environment of confinement buildings due to the high animal density^1^,^2^, which leads to poor indoor air quality. The PM contains microorganisms and endotoxins, which can cause lung infections and airway-related inflammatory responses in both farmers and animals^3^,^4^,^5^.

Culture-based methods have been predominantly used in studying airborne fungi in various animal confinement buildings^6^,^7^,^8^. The fungal colony forming units (cfu) reported in these studies range in concentration from 10^3^ cfu/m^3^ to 10^6^ cfu/m^3^, and *Cladosporium*, *Aspergillus* and *Penicillium* were detected as the predominant fungal genera. Other genera were detected, including *Alternaria*, *Fusarium*, *Verticillium*, and *Geotrichum*. It has been also found that indoor air fungal concentrations and emissions are influenced by the manure removal system in swine houses^9^. Viegas *et al*.^10^ studied air borne fungi in Portuguese swine houses and detected keratinophilic (*Scopulariopsis brevicaulis*) and toxigenic fungi (*Aspergillus*, *Fusarium*, and *Penicillium* genera and *Stachybotrys chartarum*), suggesting a potential occupational health threat to farm workers. Jo *et al*.^11^ studied airborne fungi concentrations in swine sheds and reported that the summer concentrations of total fungi and fungal genera inside the swine sheds were substantially higher than the winter values. A recent study using amplification of small subunit rRNA found *Aspergillus*-*Eurotium* as the quantitatively most important fungal group in indoor air of swine confinement facility^12^. However, the variation in diversity and community composition of airborne fungi within and between swine houses across seasons is poorly understood.

In this study, we collected aerosol samples from seven commercial swine farms in South Korea during the winter and summer seasons. The airborne fungal abundance, diversity and community composition were analyzed using culture-independent molecular methods. The present study was performed to address following questions:

1. How does the abundance, diversity and composition of airborne fungi vary in swine houses both within and between seasons?
2. What are the major microclimate variables linked to the variations in abundance, diversity and composition of airborne fungi in swine houses between seasons?
3. What are the potential allergen/pathogen related fungal genera present in swine houses, and how does their overall relative abundance vary in swine houses both within and between seasons?

## Results and Discussion

**Figure 1:**
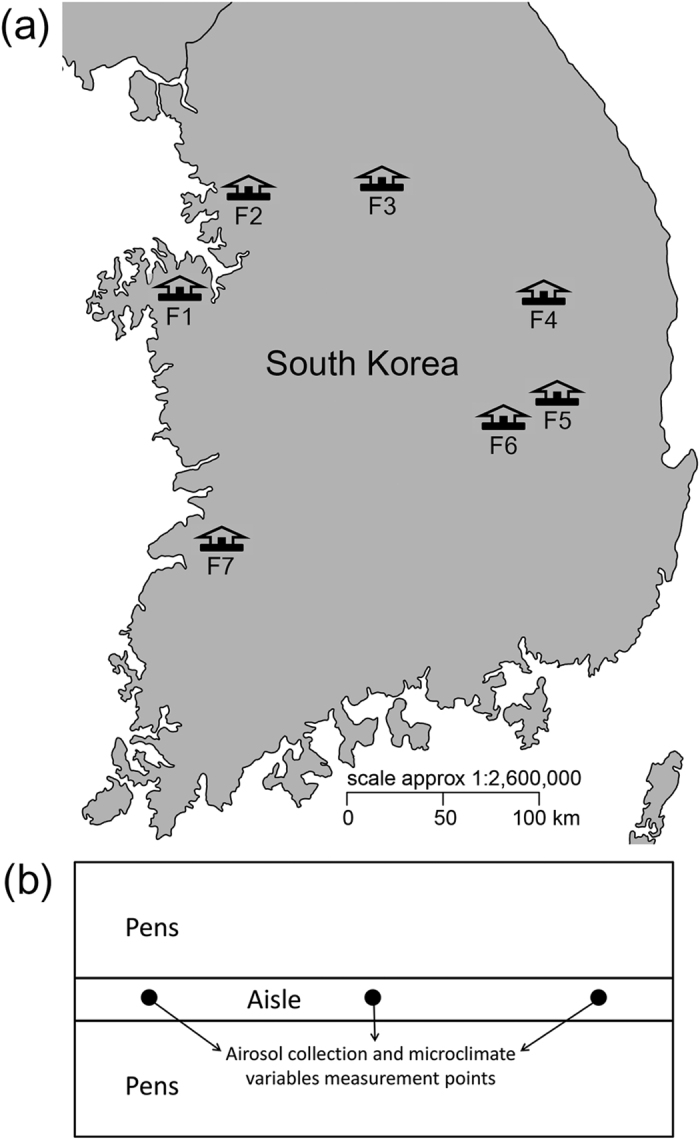
(**a**) Locations of swine farms across South Korea sampled during this study. The map was generated using ‘mapdata’ R package^62^ (https://cran.r-project.org/web/packages/mapdata/index.html). (**b**) Indoor sampling scheme diagram showing aerosol collection and microclimate variables measurement points (black circles) in swine houses.

In this study, we used quantitative PCR (qPCR) and a high-throughput Illumina sequencing to extensively examine seasonal variations in abundance, community composition and diversity of airborne fungi in seven commercial swine houses in South Korea (Fig. 1). While several studies have comprehensively investigated the airborne bacterial community composition and diversity in swine houses using next-generation sequencing (NGS) methods^13^,^14^,^15^, relatively little is known about the diversity and community composition of airborne fungi in swine houses.

### Microclimate variables

All the measured microclimate variables significantly varied between swine houses in winter (Table 1), and except PMs the other microclimate variables (temperature, relative humidity and air speed) also varied significantly between swine houses in summer (Table 1). We also found that except relative humidity, H_2_S, and stocking density, the other microclimate variables varied significantly in swine houses between seasons (Table 1).

### Total airborne fungal abundance in swine houses

**Figure 2:**
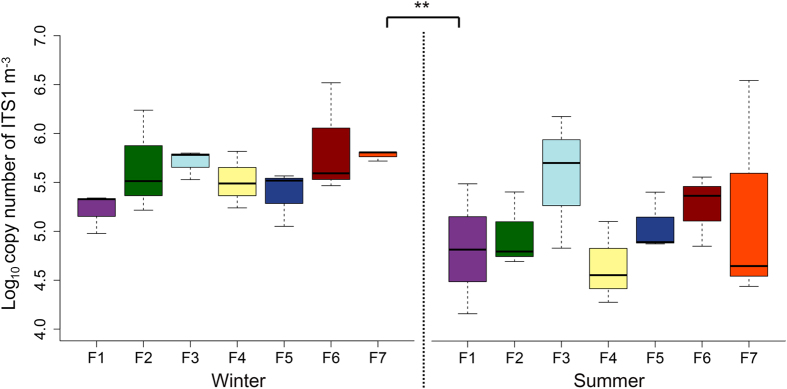
The qPCR analysis of fungal ITS1 copy numbers in aerosols samples of swine houses collected during winter and summer seasons. Double asterisk indicates statistical significant at *P* < 0.01.

The total airborne fungal abundance (universal fungal ITS primer) measured using qPCR was significantly higher in winter compared to summer (Fig. 2). However, fungal abundance did not vary between swine houses within both seasons. The bacterial abundance was also significantly higher during winter in same swine houses^14^. One of the possible explanations for this finding could be related to reduced ventilation in swine houses during winter to avoid heat loss resulted in increased concentrations of airborne particulates^16^,^17^, which could lead to increase in fungal abundance during winter. Indoor air fungal concentration was shown to increase with PMs concentrations^18^. This explanation is also supported by the results of correlation analysis, which indicate a negative correlation between fungal abundance and temperature, and airspeed (Table 2), whereas CO_2_, NH_3_, and PM2.5 showed positive correlation with fungal abundance (Table 2). However, earlier studies in swine houses based on culture-depended methods reported either higher concentrations of airborne fungi in summer^19^ or no significant difference in abundance of airborne fungi between winter and summer^20^. The discrepancy in our results from those of the previous studies could be attributed to the difference in the techniques used to measure the airborne fungal concentrations. Culture-independent qPCR method used in our study is more sensitive and accurate than conventional culture based methods for determining concentrations of total airborne microbes^21^.

### Diversity of airborne fungi in swine houses

**Figure 3:**
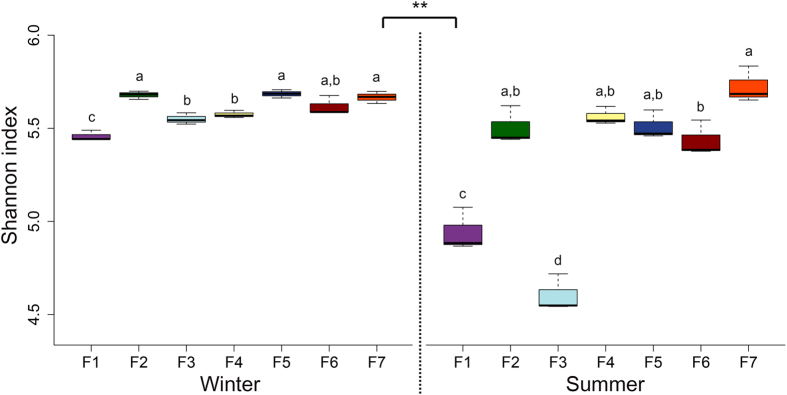
Variations in the fungal Shannon diversity index of swine houses both within and between seasons. Different letters represent statistical significant (*P* < 0.05) based on Tukey’s HSD test.

From the 42 samples, we observed 22,399 OTUs with an average 1,983 OTUs (range 1,524 to 2,345). The Shannon diversity index of airborne fungi was significantly different in swine houses both within and between seasons (Fig. 3). The Shannon diversity index was more variable between swine houses during summer compared to winter, however overall the Shannon index was significantly higher in winter compared to summer (Fig. 3). The bacterial diversity indices were also reported higher in winter season in our earlier study in same swine houses^14^. Due to the lack of studies on seasonal variation of airborne fungi in swine houses, we could not compare directly these results with others studies. However, our results are similar to the results obtained in indoor environment of residential buildings by Adams *et al*.^22^, who also observed higher fungal diversity in winter season.

We found significant positive correlation between fungal diversity and PM2.5 and PM10 concentration (Table 2), and concentration of these particles were higher in winter season (Table 1). These results together indicate one possible explanations of the high diversity in winter, which could be that to maintain the indoor air temperature during winter, all of the openings are closed and the ventilation rates are reduced to minimal, which in turn increases the indoor bioaerosol particles and thus increases microbial diversity. The indoor fungal diversity information might be useful in terms of health and exposure evaluations of animals and farmers working in swine houses, as indoor fungal diversity have been shown to be associated with asthma development^23^,^24^.

### Dominant airborne fungal taxa in swine houses

**Figure 4:**
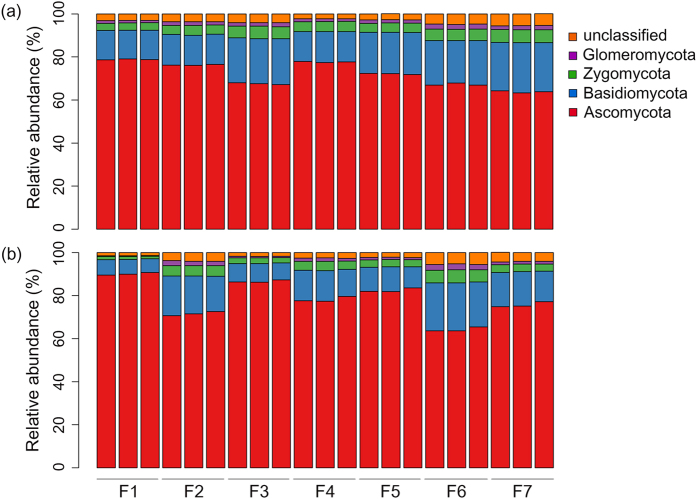
The relative abundance of airborne fungal phyla in swine houses during (**a**) winter and (**b**) summer seasons.

The most abundant fungal phyla across all of samples were Ascomycota, representing 75.4% of all sequences, followed by Basidiomycota (15.3%), Zygomycota (4.2%), and Glomeromycota (1.5%) (Fig. 4). Similar to the diversity, the relative abundance of the dominant fungal phyla also varied significantly in swine houses both within and between seasons (Table 3). The relative abundance of Ascomycota was significantly higher in summer (*P* < 0.01; Table 3), whereas the relative abundances of Basidiomycota and Zygomycota were significantly higher in winter (*P* = 0.01; Table 3). The relative abundance of Ascomycota and Basidiomycota showed negative and positive correlation, respectively with PM2.5 and PM10 (Table 2). The growth forms of Ascomycota are small enough to become easily aerosolized compared to the large growth forms of Basidiomycota^25^, this might be the explanation of dominance of Ascomycota in indoor air of swine houses across both seasons. However, the relative abundance of Ascomycota had declined in winter at expense of increase in relative abundance of Basidiomycota, which might have enriched due to high concentration of indoor airborne particles during winter.

**Figure 5:**
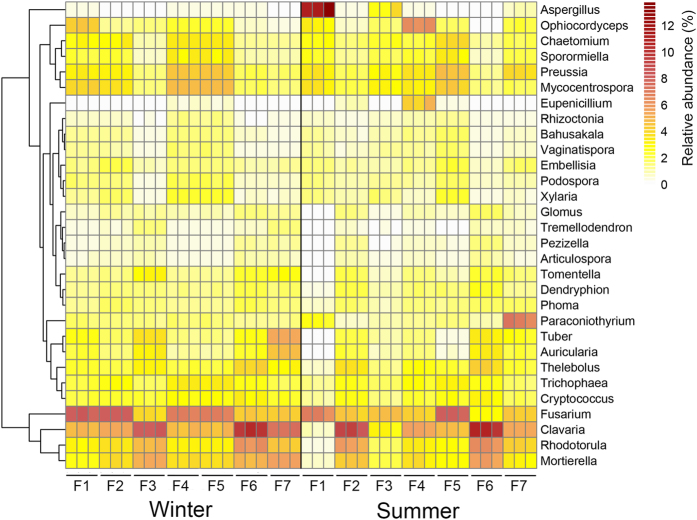
Heat map showing the relative abundance of 30 most dominant fungal genera in swine houses during winter and summer seasons.

Comparisons between swine houses were also conducted at class and genus levels. The predominant fungal classes detected in this study were *Dothideomycetes* and *Sordariomycetes* of phylum Ascomycota, which has been shown to dominate aerosol samples in several previous studies^26^,^27^,^28^. *Dothideomycetes* class is known to contain several allergenic fungal taxa^29^,^30^,^31^. *Agaricomycetes* was the most abundant class of phylum Basidiomycota, which does not generally contain described human allergenic/pathogenic fungal taxa. Although the relative abundance of all dominant fungal classes varied significantly in swine houses within seasons (Table S1), the relative abundance of only four dominant classes (*Agaricomycetes*, *Pezizomycetes*, *Eurotiomycetes*, and *Tremellomycetes*) varied significantly between seasons (Table S1). Of these, *Agaricomycetes*, *Pezizomycetes*, and *Tremellomycetes* were more abundant in winter, whereas *Eurotiomycetes* was more abundant in summer. The predominant fungal genera detected were *Clavaria* and *Fusarium*. Most of the *Clavaria* species are believed to be saprotrophic and probably originated from the litter bedding material, whereas genus *Fusarium* have been shown to dominate indoor environment of swine houses^6^,^10^,^32^. Similar to dominant fungal phyla and families, the relative abundance of 30 most dominant fungal genera also varied in swine houses both within and between seasons (Fig. 5). The relative abundance of most of the dominant fungal classes and genera were significantly correlated with the concentration of PMs and stocking density (Table S2). These results indicate the potential role of PMs concentration in structuring the airborne fungal community even at lower taxonomic levels.

### Fungal OTU composition and effect of microclimate variables

**Figure 6:**
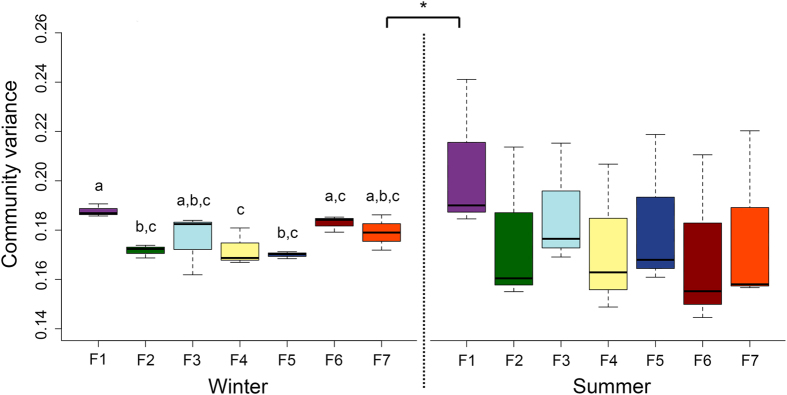
Community variance (beta diversity) of airborne fungal communities of swine houses both within and between seasons. An asterisk indicates statistical significant at *P* < 0.05, and different letters represent means that are significantly different at *P* < 0.05 (Tukey’s HSD test).

**Figure 7:**
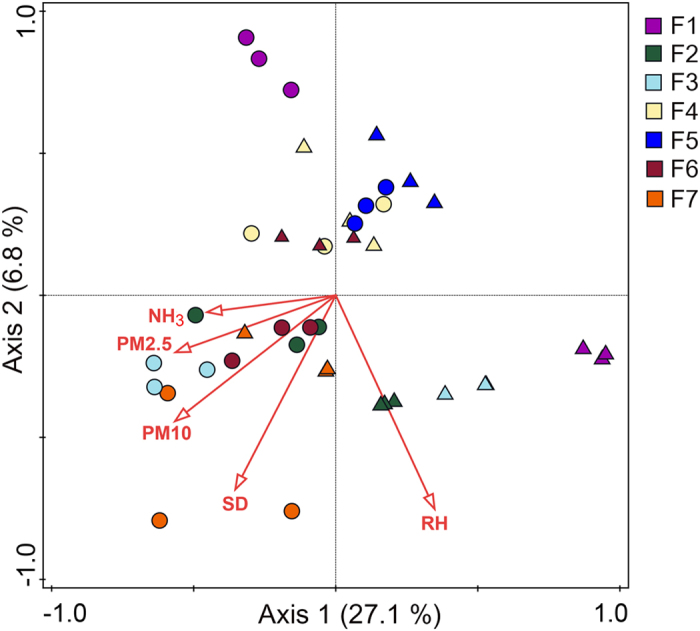
Redundancy analysis (RDA) of the association of airborne fungal community composition with microclimate variables. Circles and triangles represent samples collected during winter and summer seasons, respectively.

The PerMANOVA results showed that the airborne fungal OTU composition was influenced by both swine house unit (*F* = 4.25, *P* < 0.0001) and season (*F* = 6.25, *P* < 0.0001). The interaction between swine house unit and season also impacted significantly the fungal OTU composition (*F* = 3.5, *P* < 0.0001). Seasonal difference in bacterial OTU composition was also observed in our previous study in same swine houses^14^. Furthermore, similarly to our findings in the present study, Adams *et al*.^33^ demonstrated that both residential unit and season largely influenced the indoor airborne fungal communities. The beta diversity of indoor airborne fungi varied significantly in swine houses between seasons, with summer having significantly higher beta diversity than winter (*P* < 0.05) (Fig. 6). The variation in beta diversity among replicates within swine houses was also high during summer, which suggests that the airborne fungal community composition is more heterogeneous in summer than in winter. The RDA analysis showed that relative humidity, PMs (PM2.5 and PM10), NH_3_, and stocking density best explained the variation in community composition of airborne fungi in swine houses across seasons (Fig. 7). Relative humidity and stocking density is shown to affect the aerosolization of fungal particulates^34^,^35^,^36^, which in turn could affect the composition of airborne fungi. Whereas it has been shown that NH_3_ is one of the main precursors of secondary PMs^37^, which could then affect the community structure of airborne fungi.

### Potential Allergen/pathogen related fungal genera in swine houses

**Figure 8:**
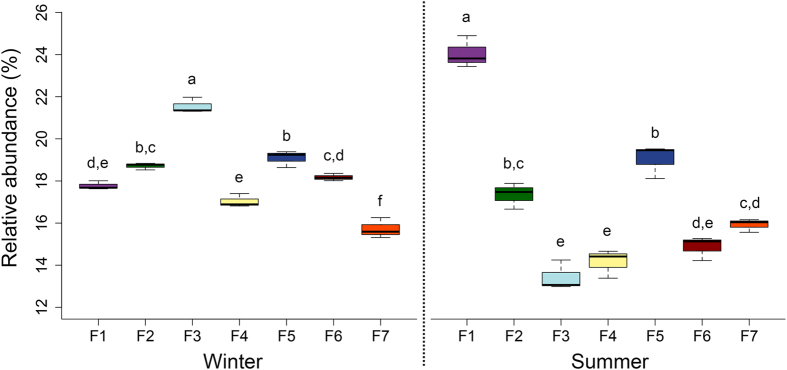
Variations in the relative abundance of potential allergen/pathogen related fungal genera between swine houses within and across seasons. Different letters represent significant differences (P < 0.05) between the treatment means (Tukey’s HSD test).

Potential allergen/pathogen related fungal genera were also examined in swine houses. A total of 80 allergen/pathogen related fungal genera are listed by Simon-Nobbe *et al*.^38^ and of these, we identified a total of 29 potential allergen/pathogen related fungal genera. The relative abundance of total potential allergen/pathogen related fungal genera varied from 13.0–24.9%. Overall, the relative abundance of potential allergen/pathogen related fungal genera varied significantly between swine houses during each season (Fig. 8), however their relative abundance did not vary significantly between seasons (Fig. 8). The most abundant potential human allergen/pathogen related fungal genus detected was *Fusarium* (10.8%). *Fusarium* is an emerging fungal pathogen and can cause infections in humans, especially in immunocompromised hosts^39^,^40^. The relative abundance of potential allergen/pathogen related fungal genera was positively correlated with PM2.5 concentrations (Table 2). The potential allergen/pathogen related genera deposited on PM2.5 could cause respiratory problems in both farm workers and animals, as PM2.5 can penetrate and deposit deeper in the tracheobronchial and alveolar regions^41^. These results, however, should be taken with the caveat that the taxonomy of an organism does not necessarily provide the information about the allergenic/pathogenic level of that organism, so the information reported here on allergenicity/ pathogenicity of fungal genera is solely of ‘potential’ character.

## Conclusions

In conclusion, our results suggest that the swine house unit and season and their interaction influenced the diversity and community composition of airborne fungi. The indoor air fungal abundance and diversity were significantly higher in winter than in summer. The abundance, diversity and composition of airborne fungi were significantly correlated with microclimate variables, mainly by the relative humidity, PMs, and stocking density. Several potential allergen/pathogen related fungal genera were also observed, and their total relative abundance varied between swine houses in both seasons. These potential allergen/pathogen related fungal genera present in the indoor air of swine houses and their association with PM2.5 could impact the health of farm workers and animals. Overall, this study provides a better understanding of abundance, diversity and community composition of airborne fungi in swine houses across seasons.

## Methods

### Characteristics of swine houses and aerosol collection

Aerosol samples were collected from seven commercial swine farms in South Korea in winter (January) and summer (June) of 2013, with prior permissions from farm owners. The aerosol sampling was performed in growing/finishing swine houses equipped with mechanical ventilation system. The number of animals in sampled houses varied from 140 to 480 with the stocking density ranged from 0.88 to 1.41 m^2^/head. All the swine houses were equipped with deep-pit manure removal with slats.

We collected aerosol samples at a height of 1.4 m above the ground from three points in swine houses. Autoclaved cellulose nitrate filters (0.22 μm; Fisher Scientific, Pittsburgh, PA) were used to collect the aerosol samples via filtration with a constant flow rate of approximately 4 L min^−1^ for 24 h. After aerosol collection, the filters were immediately transported to the laboratory, where the samples were frozen at −20 °C.

### Microclimate variables

The microclimate variables were measured several times during the sampling period from the same three aerosol collection points, and the average values were reported corresponding to each sampling point. A hygrothermograph (SK-110TRH, SATO, Tokyo, Japan) was used to measure the air temperature and relative humidity. We used an anemometer (model 6112, KANOMAX, Osaka, Japan) to measure the air speed. An aerosol mass monitor (GT-331, SIBATA, Soca-city, Japan) was used to measure the concentrations of PM2.5 (mean aerodynamic diameter ≤2.5 μm) and PM10 (average aerodynamic diameter ≤10 μm). Gas detector tubes (Gastec Co., Ltd., Kanagawa, Japan) were used to measure the concentrations of ammonia (NH_3_), hydrogen sulfide (H_2_S) and carbon dioxide (CO_2_).

### DNA extraction and universal fungal qPCR

The PowerSoil DNA isolation kit (MoBio Laboratories, Carlsbad, CA) was used to extract DNA from filters by following the initial processing methods as described in Kumari *et al*.^14^. The purified DNA samples were used for quantitative PCR (qPCR) analysis to quantify the copy numbers of fungal ITS. The ITS1 region was targeted with the universal fungal primers ITS1F and ITS2^42^,^43^. Each of 20 μL reaction mixtures contained 1× Fast SYBR Green Master mix reagent (Clontech Laboratories, Inc., Mountain View, CA, USA), 10 μM forward and reverse primers, and 1 μL of template DNA. Quantitative PCR was performed on the 7300 Real-Time PCR System (Applied Biosystems, Inc., Foster City, CA, USA). Amplification was initiated with a 15 min denaturation at 95 °C, followed by 45 cycles of a 15 sec dissociation at 95 °C and a 1 min annealing and extension at 60 °C. Standard curves were generated based on a standard ITS1 amplicon with a known copy number concentration. The standard amplicon was prepared with conventional PCR using the universal primers ITS1F/ITS2 and DNA extract from *Aspergillus fumigatus* ATCC MYA4609 as a template. The concentration of the standard amplicon was quantitated by the Quant-iT PicoGreen dsDNA reagent kit (Life Technologies, Carlsbad, CA, USA). The amplicon was serially diluted from 1 to 10^6^ copy number μL^−1^ to calibrate qPCR. Each qPCR measurement was in triplicate. PCR inhibition was checked with the method reported elsewhere^44^. No inhibition was found. In this study, 10% of DNA extraction efficiency was assumed to calculate copy number per 1 m^3^ of air^44^, though the efficiencies might be different across the studies since the different extraction methods were used. However, the comparison was possible for the samples collected within this study since the same extraction method was used for all the samples collected within this study.

### PCR amplification and sequencing

We amplified the ITS1 region using primer pairs ITS1FI2, 5′-GAACCWGCGGARGGATCA-3′^45^ and ITS2, 5′-GAACCWGCGGARGGATCA-3′^43^. Amplifications were carried out in a total volume of 30 μL composed of 1 or 2 μL of template DNA, 1× PCR Master Mix (Takara Bio, Shiga, Japan), and 0.3 μM of each primer. To account for the stochasticity of PCR reactions amplification was performed at two annealing temperatures (52 °C and 55 °C) in three replicates for each annealing temperature^45^. Schmidt *et al*.^45^ suggested that using multiple annealing temperatures could decrease the primer binding bias, and enable the recovery of more complete fungal communities. PCR was carried out under the following conditions: initial denaturation step for 15 min at 95 °C, followed by 30 cycles of 30 sec at 95 °C, 30 sec at either 52 °C or 55 °C, and 30 sec at 72 °C, and a final elongation step for 5 min at 72 °C. The DNA extracted from sterilized filters kept in −20 °C was served as negative controls. The negative controls did not show any amplification. The amplicons were purified using the QIAquick PCR purification kit (Qiagen, CA, USA), and sent to the Beijing Genome Institute (BGI) (Hong Kong, China) for sequencing using 2 × 150 bp Hiseq2000 platform (Illumina, San Diego, CA, USA).

### Sequence processing

The paired-end ITS1 sequences were assembled using Pandaseq software^46^, followed by removal of the flanking rRNA gene fragments from ITS1 region using ITSx version 1.0.9^47^. The ITS1 sequences were further processed in mother version 1.36.1^48^. The chimeric ITS1 sequences were detected and removed with mothur’s implementation of uchime in *de novo* mode^49^. Taxonomic assignments of ITS1 sequences were performed using BLASTn ver. 2.2.19^50^ against a named fungal ITS sequences database^51^, and further classified using a fungal taxonomic identification tool FHiTINGS^52^. The sequences were not clustered into operational taxonomic units (OTUs) before taxonomic assessments as clustering process may reduce the taxonomic coverage^53^. The taxonomic identifications of ITS sequences are uncertain at the species level^54^, therefore the taxonomic identifications were performed only down to the genus level. The potential allergen/pathogen related fungal genera were identified using a list of known fungal allergen/pathogen related fungal genera^27^. The ITS1 sequences were clustered into OTUs with a threshold of 97% sequence similarity using the QIIME implementation of UCLUST^55^. All sequence data are deposited in the MG-RAST server^56^ under MG-RAST IDs 4633146.3–4633187.3.

### Statistical analysis

Prior to the calculation of diversity indices, all the samples were rarified to 24,000 reads per sample by random subsampling using the ‘sub.sample’ command in mothur. The Bray-Curtis distance was used to calculate the OTU-based community dissimilarity^57^. The differences in microclimate variables, fungal abundance, OTU richness, Shannon index and the relative abundance of the most abundant phyla between swine houses within seasons were analyzed using ANOVA followed by Tukey’s HSD test with Benjamini and Hochberg correction to adjust the p-values^58^. Adjusted p-values of less than 0.05 were considered statistically significant. Furthermore, the seasonal differences in these parameters were evaluated using t-test. We performed Spearman rank correlation to test the relationship between microclimate variables (temperature, relative humidity, air speed, NH_3_, H_2_S, CO_2_ and stocking density) and microbial measurements (fungal abundance, diversity index and relative abundance of fungal taxa). A heatmap of the 30 most abundant fungal genera was constructed using the ‘pheatmap’ package in R.

To test how the fungal community composition is influenced by the swine house unit and season we performed a permutational multivariate analysis of variance test (PerMANOVA, ‘adonis’ function in ‘vegan’ R package^59^. We used redundancy analysis (RDA) to test which microclimate variables best explain the variation in fungal community composition using Canoco 5.0 (Biometrics, Wageningen, The Netherlands), applying forward selection and the Monte Carlo permutation test with 999 random permutations. We also performed a permutational dispersion analysis to test whether beta diversity is significantly different in swine houses both within and between seasons by using the betadisper function in the ‘vegan’ R package^60^. All statistical analysis, graphs, and ordinations were produced using R version 3.0.2^61^.

## Additional Information

**How to cite this article**: Kumari, P. *et al*. Variations in abundance, diversity and community composition of airborne fungi in swine houses across seasons. *Sci. Rep.* **6**, 37929; doi: 10.1038/srep37929 (2016).

**Publisher's note:** Springer Nature remains neutral with regard to jurisdictional claims in published maps and institutional affiliations.

## Supplementary Material

Supplementary Information

## Figures and Tables

**Table 1:** The microclimate variables (mean ± SD) measured in swine houses both within and between seasons.

Farm	Season	Temperature (°C)^1^	Relative humidity (%)	Air speed (m/s)	PM10 (μg m^−3^)	PM2.5 (μg m^−3^)	NH_3_ (mg L^−1^)	H_2_S (mg L^−1^)	CO_2_ (mg L^−1^)	Stocking Density (m^2^ head^−1^)
F1	Winter	16.6 ± 0.2^c^	48.3 ± 4.1^d^	0.00 ± 0.00^b^	207.9 ± 74.7^b,c^	42.8 ± 5.4^b,c^	6.5 ± 3.0^c^	0.0 ± 0.0^c^	1483.3 ± ± 125.8^c^	0.94
F2		19.3 ± 0.6^d^	92.0 ± 0.6^a,b^	0.00 ± 0.00^b^	214.0 ± 219.3^b,c^	105.5 ± 86.7^b^	40.4 ± 9.3^a,b^	0.9 ± 0.3^a^	2975.0 ± 438.0^b^	1.15
F3		21.8 ± 0.1^b^	98.6 ± 1.3^a^	0.02 ± 0.01^a,b^	665.9 ± 31.4^a,b^	233.0 ± 12.6^a^	68.2 ± 8.9^a^	0.1 ± 0.0^c^	3243.3 ± 94.5^b^	1.14
F4		25.7 ± 1.3^a^	76.3 ± 1.7^b,c^	0.02 ± 0.00^a,b^	23.8 ± 11.6^c^	17.3 ± 9.7^c^	43.9 ± 23.1^a,b^	0.3 ± 0.2^b,c^	3190.0 ± 208.1^b^	0.93
F5		25.0 ± 0.4^a^	81.2 ± 2.5^a,b,c^	0.04 ± 0.00^a^	189.5 ± 108.4^b,c^	128.9 ± 58.1^a,b^	32.3 ± 12.5^b,c^	0.3 ± 0.2^b,c^	3300.0 ± 217.9^b^	0.88
F6		25.2 ± 0.2^a^	67.8 ± 6.2^c^	0.02 ± 0.01^a,b^	1216.7 ± 335.5^a^	45.7 ± 6.2^b,c^	59.1 ± 5.3^a,b^	0.7 ± 0.2^a,b^	4766.7 ± 1118.4^a^	0.91
F7		21.1 ± 1.2^b,c^	81.9 ± 16.1^a,b,c^	0.04 ± 0.02^a^	1397.3 ± 730.9^a^	103.7 ± 13.4^a,b^	7.5 ± 2.5^c^	0.0 ± 0.0^c^	2816.7 ± 35.1^b^	1.41
F1	Summer	30.2 ± 0.8^c^	99.0 ± 1.1^a^	0.16 ± 0.02^b^	63.5 ± 5.1	7.8 ± 0.2	10.8 ± 2.3^c,d^	0.0 ± 0.0^c^	1136.7 ± 125.0^c,d^	0.90
F2		26.6 ± 0.3^d^	100.0 ± 0.0^a^	0.12 ± 0.04^b^	79.3 ± 20.0	34.1 ± 3.2	5.5 ± 1.8^d^	0.1 ± 0.2^c^	1026.7 ± 33.3^c,d^	1.29
F3		26.6 ± 0.3^d^	100.0 ± 0.0^a^	0.12 ± 0.04^b^	68.6 ± 19.5	11.6 ± 1.2	19.3 ± 7.5^b,c^	0.5 ± 0.1^b,c^	1712.5 ± 119.2^a,b^	0.96
F4		38.5 ± 0.9^a^	71.4 ± 8.7^b^	0.13 ± 0.05^b^	101.6 ± 45.0	18.2 ± 5.2	27.7 ± 6.3^a,b^	1.1 ± 0.0^a^	1886.7 ± 168.1^a^	0.95
F5		35.7 ± 0.5^b^	68.9 ± 5.9^b^	0.51 ± 0.24^a^	52.7 ± 13.2	16.5 ± 2.5	6.2 ± 2.5^d^	0.3 ± 0.1^b,c^	850.8 ± 78.9^d^	0.93
F6		31.4 ± 0.3^c^	76.1 ± 1.0^b^	0.20 ± 0.07^a,b^	71.6 ± 22.6	15.7 ± 1.2	33.2 ± 5.5^a^	0.8 ± 0.5^a,b^	1316.7 ± 246.6^b,c^	1.03
F7		31.7 ± 1.2^c^	88.5 ± 4.3^a^	0.25 ± 0.02^a,b^	105.4 ± 15.9	16.2 ± 5.0	1.6 ± 0.4^d^	0.0 ± 0.0^c^	768.3 ± ± 164.3^d^	1.41
*P*-value^2^		0.002	0.13	<0.0001	0.0002	<0.0001	0.001	0.63	<0.0001	0.79

^1^Means with SD on the same row with different letters denote significant differences (*P* < 0.05) based on Tukey’s HSD test with Benjamini–Hochberg correction for multiple comparisons.

^2^For each variable, *P*-value was used to determine the significance of means across seasons.

**Table 2:** Spearman rank correlations between microclimate variables and diversity indices, the relative abundance of fungal phyla and pathogen/allergen related genera.

	Temperature	Relative humidity	Air speed	PM10	PM2.5	NH_3_	H_2_S	CO_2_	Stocking Density
**Fungal abundance**	−0.45**	0.07	−0.39*	0.24	0.34*	0.34*	0.12	0.47**	0.11
**Fungal diversity**									
OTU richness	−0.48**	−0.21	−0.40*	0.52***	0.49**	0.06	−0.18	0.27	0.01
Shannon index	−0.26	−0.12	−0.25	0.43**	0.55***	0.08	0.07	0.33*	0.21
**Fungal phyla**									
Ascomycota	0.21	0.15	0.1	−0.33*	−0.34*	−0.30	−0.03	−0.36*	0.30
Basidiomycota	−0.24	−0.12	−0.12	0.35*	0.38*	0.33*	0.00	0.40*	−0.21
Zygomycota	−0.23	−0.08	−0.18	0.21	0.26	0.32*	−0.02	0.41*	−0.28
Glomeromycota	−0.14	−0.07	−0.07	0.2	0.15	0.27	0.08	0.30	−0.42*
**Pathogen/allergen related genera**	−0.29	0.07	−0.23	0.16	0.32*	0.20	−0.23	0.18	−0.43*

^∗^*P* < 0.05; ^∗∗^*P* < 0.01; ^∗∗∗^*P* < 0.001.

**Table 3:** The relative abundance of fungal phyla (mean ± SD) detected in indoor air of swine houses both within and between seasons.

Farm	Season	Ascomycota^1^	Basidiomycota	Zygomycota	Glomeromycota
F1	Winter	78.8 ± 0.2^a^	13.5 ± 0.1^d^	3.4 ± 0.1^d^	1.1 ± 0.1^e^
F2		76.2 ± 0.3^c^	14.1 ± 0.2^d^	4.3 ± 0.2^c^	1.6 ± 0.2^b,c^
F3		67.5 ± 0.4^e^	21.1 ± 0.2^b^	5.6 ± 0.2^b^	1.7 ± 0.1^b,c^
F4		77.7 ± 0.3^b^	14.1 ± 0.3^d^	4.6 ± 0.1^c^	1.3 ± 0.1^d,e^
F5		72.1 ± 0.3^d^	19.2 ± 0.2^c^	4.3 ± 0.1^c^	1.5 ± 0.1^c,d^
F6		67.2 ± 0.6^e^	20.3 ± 0.6^b^	5.3 ± 0.1^b^	2.3 ± 0.1^a^
F7		63.8 ± 0.5 ^f^	22.9 ± 0.4^a^	6.0 ± 0.0^a^	1.9 ± 0.2^b^
F1	Summer	90.0 ± 0.6^a^	6.8 ± 0.4^e^	1.4 ± 0.1 ^f^	0.3 ± 0.1^e^
F2		71.6 ± 1.0^e^	17.5 ± 1.0^b^	4.8 ± 0.1^b^	2.2 ± 0.2^b^
F3		86.6 ± 0.6^b^	8.3 ± 0.5^e^	2.6 ± 0.1^e^	0.7 ± 0.1^e^
F4		78.2 ± 1.2^d^	13.6 ± 0.9^c^	4.1 ± 0.3^c^	1.5 ± 0.2^c^
F5		82.4 ± 1.0^c^	10.8 ± 0.9^d^	3.4 ± 0.1^d^	1.1 ± 0.1^d^
F6		64.2 ± 1.0^f^	21.8 ± 0.8^a^	5.9 ± 0.2^a^	2.6 ± 0.1^a^
F7		75.7 ± 1.3^d^	15.4 ± 1.0^b,c^	3.5 ± 0.2^d^	1.2 ± 0.0^c,d^
*P*-value^2^		0.006	0.002	0.003	0.15

^1^Tukey’s HSD test with Benjamini–Hochberg correction was used for multiple comparisons, and values (mean ± SD) on the same column with different letters denote significant differences (*P* < 0.05).

^2^*P*-value was used to determine the significance of means of each variable across seasons.
