# Supplementary material for: Variations in abundance, diversity and community composition of airborne fungi in swine houses across seasons
Source: Sci Rep. 2016 Nov 28;6:37929. doi: 10.1038/srep37929 (PMC5124938; doi:10.1038/srep37929)
Supplement: Supplementary Information [file srep37929-s1.doc]

**Supplementary Online Material**

**Variations in abundance, diversity and community composition of airborne fungi in swine houses across seasons**

Priyanka Kumari^1^*, Cheolwoon Woo^2^, Naomichi Yamamoto^2^, and Hong-Lim Choi^1^*****

^1^Department of Agricultural Biotechnology, Research Institute for Agriculture and Life Science, Seoul National University, Seoul 151-921, South Korea.

^2^Department of Environmental Health, Graduate School of Public Health, Seoul National University, Seoul 151-742, South Korea.

*Corresponding Authors:

Priyanka Kumari, Tel: +82-2-880-4821, Fax: +82-2-874-4808, E-mail: priyanka.shahi1983@gmail.com

Hong-Lim Choi, Tel: +82-2-880-4808, Fax: +82-2-874-4808,

E-mail: [ulsoo8@snu.ac.kr](mailto:ulsoo8@snu.ac.kr)

Running title: Airborne fungal diversity and composition in swine houses

**Table S1.** The relative abundance of dominant fungal classes (mean±SD) in aerosol samples of swine houses between seasons.

| Farm | Season | *Dothideomycetes^1^* | *Sordariomycetes* | *Agaricomycetes* | *Leotiomycetes* | *Pezizomycetes* | *Microbotryomycetes* | *Eurotiomycetes* | *Tremellomycetes* | *Glomeromycetes* |
| --- | --- | --- | --- | --- | --- | --- | --- | --- | --- | --- |
| F1 | Winter | 25.4±0.3^c^ | 32.6±0.5^a^ | 6.8±0.1^d^ | 5.1±0.1^d^ | 5.0±0.1^d^ | 3.5±0.1^c^ | 2.5±0.0^c^ | 3.0±0.0^c^ | 1.1±0.1^c^ |
| F2 |  | 27.2±0.3^b^ | 27.7±0.2^b^ | 7.1±0.2^d^ | 5.9±0.1^c^ | 5.0±0.1^d^ | 3.9±0.2^c^ | 2.6±0.1^b,c^ | 2.9±0.0^c^ | 1.6±0.2^b^ |
| F3 |  | 16.7±0.1^f^ | 17.1±0.2^e^ | 11.7±0.2^b^ | 7.7±0.1^b^ | 5.8±0.1^b^ | 5.6±0.2^b^ | 1.7±0.1^e^ | 3.2±0.3^b,c^ | 1.7±0.1^b^ |
| F4 |  | 31.1±0.2^a^ | 26.9±0.2^c^ | 6.9±0.2^d^ | 4.8±0.1^d^ | 5.2±0.1^d^ | 3.5±0.1^c^ | 2.7±0.1^b^ | 3.4±0.1^b^ | 1.3±0.1^c^ |
| F5 |  | 31.1±0.2^a^ | 26.9±0.2^c^ | 6.9±0.2^d^ | 4.8±0.1^d^ | 5.2±0.1^d^ | 3.5±0.1^c^ | 2.7±0.1^b^ | 3.4±0.1^b^ | 1.3±0.1^c^ |
| F6 |  | 20.1±0.2^d^ | 16.8±0.2^e^ | 9.1±0.2^c^ | 9.3±0.1^a^ | 5.5±0.0^c^ | 6.8±0.3^a^ | 3.1±0.1^a^ | 4.1±0.2^a^ | 2.3±0.1^a^ |
| F7 |  | 17.5±0.2^e^ | 18.9±0.3^d^ | 12.9±0.4^a^ | 7.5±0.3^b^ | 7.8±0.2^a^ | 5.4±0.1^b^ | 2.0±0.0^d^ | 4.3±0.1^a^ | 1.9±0.2^b^ |
| F1 | Summer | 23.8±0.4^d^ | 26.1±0.7^b^ | 2.2±0.1^e^ | 2.2±0.1^f^ | 1.9±0.1^e^ | 0.5±0.0^g^ | 14.3±1.6^a^ | 1.2±0.2^e^ | 0.3±0.1^e^ |
| F2 |  | 21.3±0.5^e^ | 19.5±0.5^d,e^ | 7.9±0.5^b,c^ | 8.2±0.2^b^ | 4.9±0.3^b^ | 6.0±0.4^b^ | 2.8±0.2^c,d^ | 3.3±0.2^b^ | 2.2±0.2^b^ |
| F3 |  | 19.7±0.3^f^ | 18.4±0.3^e^ | 4.0±0.2^d^ | 3.1±0.2^e^ | 3.1±0.2^d^ | 2.0±0.1^f^ | 4.9±0.9^b,c^ | 1.7±0.1^e^ | 0.7±0.1^e^ |
| F4 |  | 26.6±0.2^c^ | 23.0±0.0^c^ | 7.1±0.5^c^ | 6.4±0.3^c^ | 4.6±0.3^b,c^ | 3.7±0.2^d^ | 6.7±0.8^b^ | 2.6±0.2^c,d^ | 1.5±0.2^c^ |
| F5 |  | 33.3±0.4^a^ | 29.0±0.2^a^ | 4.6±0.4^d^ | 5.2±0.4^d^ | 3.9±0.2^c^ | 2.9±0.2^e^ | 2.2±0.2^d^ | 3.1±0.1^b,c^ | 1.1±0.1^d^ |
| F6 |  | 17.9±0.7^g^ | 16.0±0.6^f^ | 10.3±0.2^a^ | 9.6±0.5^a^ | 6.2±0.3^a^ | 7.0±0.4^a^ | 1.4±0.1^d^ | 4.1±0.1^a^ | 2.6±0.1^a^ |
| F7 |  | 30.9±0.2^b^ | 20.1±0.5^d^ | 8.2±0.5^b^ | 5.3±0.2^d^ | 5.9±0.4^a^ | 4.6±0.2^c^ | 2.9±0.5^c,d^ | 2.3±0.0^d^ | 1.2±0.0^c,d^ |
| *P*-value^2^ |  | 0.72 | 0.19 | 0.004 | 0.27 | 0.002 | 0.16 | 0.01 | 0.001 | 0.23 |

^1^ *D*ifferent letter in same column indicates statistical significance at *P* < 0.05 (Tukey’s HSD test).

^2^ *P*-value was used to determine the significance of means of each variable across seasons.

**Table S2.** Correlations between microclimate variables and relative abundance of dominant fungal classes and genera.

|  | Temp. | Relative  humidity | Air  speed | PM10 | PM2.5 | NH_3_ | H_2_S | CO_2_ | Stocking  Density |
| --- | --- | --- | --- | --- | --- | --- | --- | --- | --- |
| **Dominant fungal classes** |  |  |  |  |  |  |  |  |  |
| *Dothideomycetes* | 0.26 | -0.31 | 0.13 | -0.40* | -0.19 | -0.25 | 0.07 | -0.21 | -0.36 |
| *Sordariomycetes* | -0.14 | -0.30 | -0.20 | -0.26 | -0.03 | -0.29 | -0.14 | -0.18 | -0.32 |
| *Agaricomycetes* | -0.21 | -0.03 | -0.16 | 0.58*** | 0.56*** | 0.21 | -0.03 | 0.30 | 0.56 |
| *Leotiomycetes* | -0.06 | -0.15 | -0.06 | 0.42* | 0.45** | 0.19 | 0.22 | 0.15 | 0.44 |
| *Pezizomycetes* | -0.28 | -0.18 | -0.19 | 0.50** | 0.50** | 0.16 | -0.13 | 0.30 | 0.42 |
| *Microbotryomycetes* | -0.13 | -0.05 | -0.11 | 0.46** | 0.45** | 0.22 | 0.12 | 0.21 | 0.49 |
| *Eurotiomycetes* | 0.30 | 0.21 | 0.15 | -0.21 | -0.48** | -0.08 | 0.11 | -0.06 | -0.35 |
| *Tremellomycetes* | -0.28 | -0.32* | -0.25 | 0.33* | 0.49** | 0.26 | 0.08 | 0.40* | 0.04 |
| *Glomeromycetes* | -0.15 | -0.07 | -0.17 | 0.40* | 0.48** | 0.31 | 0.25 | 0.31* | 0.39 |
| **Dominant fungal genera** |  |  |  |  |  |  |  |  |  |
| *Clavaria* | -0.09 | -0.10 | -0.11 | 0.45** | 0.46** | 0.26 | 0.21 | 0.21 | 0.47** |
| *Fusarium* | -0.24 | -0.21 | -0.23 | -0.23 | -0.06 | -0.23 | -0.12 | -0.09 | -0.39* |
| *Rhodotorula* | -0.12 | -0.03 | -0.11 | 0.45** | 0.44** | 0.23 | 0.14 | 0.23 | 0.48** |
| *Mortierella* | -0.19 | -0.06 | -0.19 | 0.43** | 0.57*** | 0.39* | 0.17 | 0.44** | 0.35* |
| *Preussia* | 0.28 | -0.25 | 0.15 | -0.41* | -0.29 | -0.22 | -0.01 | -0.16 | -0.48** |
| *Mycocentrospora* | -0.01 | -0.25 | -0.17 | -0.40* | -0.16 | -0.06 | 0.07 | 0.03 | -0.62*** |
| *Thelebolus* | 0.01 | -0.27 | -0.02 | 0.40* | 0.40* | 0.17 | 0.13 | 0.14 | 0.28 |
| *Trichophaea* | 0.04 | -0.43** | -0.17 | -0.22 | 0.22 | 0.29 | 0.50** | 0.30 | -0.34* |
| *Cryptococcus* | -0.02 | -0.38* | -0.11 | 0.06 | 0.31 | 0.33* | 0.36* | 0.35* | -0.19 |
| *Chaetomium* | 0.04 | -0.21 | -0.06 | -0.48** | -0.22 | -0.18 | 0.07 | -0.11 | -0.50** |
| *Tuber* | -0.40* | -0.11 | -0.28 | 0.63*** | 0.57*** | 0.07 | -0.19 | 0.19 | 0.62*** |
| *Ophiocordyceps* | 0.18 | -0.22 | 0.01 | -0.23 | -0.30 | -0.11 | -0.11 | -0.11 | -0.49** |
| *Sporormiella* | 0.22 | -0.17 | 0.09 | -0.44** | -0.28 | -0.17 | 0.04 | -0.12 | -0.56*** |
| *Auricularia* | -0.34* | -0.12 | -0.25 | 0.60*** | 0.54*** | 0.11 | -0.13 | 0.21 | 0.58*** |
| *Paraconiothyrium* | -0.04 | -0.23 | -0.02 | -0.06 | -0.10 | -0.25 | -0.42** | -0.17 | -0.30 |
| *Tomentella* | -0.24 | -0.03 | -0.20 | 0.60*** | 0.58*** | 0.23 | 0.00 | 0.29 | 0.57*** |
| *Dendryphion* | 0.10 | -0.19 | 0.05 | 0.25 | 0.35* | 0.25 | 0.32* | 0.18 | 0.25 |
| *Phoma* | 0.05 | -0.14 | 0.05 | 0.31 | 0.29 | 0.03 | 0.22 | -0.02 | 0.52*** |
| *Embellisia* | 0.08 | -0.23 | 0.03 | -0.24 | -0.08 | -0.25 | -0.02 | -0.15 | -0.21 |
| *Aspergillus* | 0.27 | 0.07 | 0.23 | -0.27 | -0.62*** | -0.34* | -0.14 | -0.32* | -0.33* |
| *Podospora* | -0.02 | -0.21 | -0.10 | -0.43** | -0.19 | -0.18 | -0.01 | -0.07 | -0.54*** |
| *Xylaria* | 0.02 | -0.07 | -0.05 | -0.50** | -0.22 | -0.12 | 0.07 | -0.06 | -0.50** |
| *Bahusakala* | 0.15 | -0.23 | -0.01 | -0.43** | -0.19 | -0.06 | 0.16 | -0.03 | -0.57*** |
| *Glomus* | -0.14 | -0.06 | -0.17 | 0.39* | 0.48** | 0.33* | 0.28 | 0.32* | 0.39* |
| *Vaginatispora* | 0.17 | -0.29 | 0.05 | -0.47** | -0.29 | -0.23 | 0.05 | -0.16 | -0.57*** |
| *Pezizella* | -0.04 | -0.10 | -0.03 | 0.41* | 0.42** | 0.08 | 0.11 | 0.08 | 0.56*** |
| *Rhizoctonia* | 0.19 | -0.26 | 0.01 | -0.51*** | -0.22 | -0.07 | 0.21 | -0.06 | -0.51** |
| *Articulospora* | -0.03 | -0.12 | -0.03 | 0.40* | 0.43** | 0.19 | 0.21 | 0.15 | 0.46** |
| *Tremellodendron* | -0.27 | 0.06 | -0.22 | 0.55*** | 0.59*** | 0.32* | 0.07 | 0.37* | 0.59*** |
| *Eupenicillium* | 0.33 | -0.14 | 0.08 | -0.36* | -0.17 | -0.03 | 0.23 | -0.03 | -0.47** |

^∗^*P*<0.05; ^∗∗^*P*<0.01; ^∗∗∗^*P*<0.001.
